# Changes in neutrophil-lymphocyte and platelet-lymphocyte ratios before and after percutaneous coronary intervention and their impact on the prognosis of patients with acute coronary syndrome

**DOI:** 10.1016/j.clinsp.2023.100221

**Published:** 2023-07-04

**Authors:** Edenilson de Souza Teixeira, João Gabriel Ferreira de Oliveira, Rodrigo Mendes, Cristian Rodrigues do Nascimento, Johnnatas Mikael Lopes, Pedro Pereira Tenório

**Affiliations:** a Universidade Federal do Vale do São Francisco (UNIVASF), Faculdade de Medicina, Paulo Afonso, BA, Brazil; b Irmandade Santa Casa de Misericórdia de São Paulo, SP, Brazil

The authors read the article with great enthusiasm: “Changes in the neutrophil-to-lymphocyte and platelet-to-lymphocyte ratios before and after percutaneous coronary intervention and their impact on the prognosis of patients with acute coronary syndrome”, which aimed to evaluate Neutrophil/Lymphocyte (NLR) and Platelet/Lymphocyte (PLR) ratios before and after percutaneous coronary intervention (PCI) to estimate major adverse cardiac events (MACE).^1^

It is known that NLR has been a special marker of interest due to its role in the prognosis after Acute Coronary Syndrome (ACS), Systemic Arterial Hypertension (SAH), Chronic Kidney Disease (CKD), Diabetes Mellitus (DM), Heart Failure (HF), cerebrovascular disease and arterial disease. The plausible pathophysiological mechanism for this relationship is the role of neutrophils in mediating the inflammatory response to acute myocardial injury, causing further tissue damage.^2^ Other studies have shown that NLR is related to in-hospital and long-term cardiovascular mortality in patients with acute myocardial infarction (AMI) with ST-Segment Elevation (STEMI), in addition to the drop in ejection fraction after AMI, which can be a cause of HF.^3^^,^^4^

The present study demonstrated an increase in the proportion of neutrophils and NLR in patients with acute coronary syndrome (ACS) after PCI, accompanied by a reduction in the count and proportion of lymphocytes, as well as presenting a greater predictive value for the incidence of the main MACE compared to the PLR after 24 h.^1^ This finding is noteworthy, as previous studies have focused mainly on the pre-PCI NLR relationship as a significant influencing factor on the long-term prognosis of patients with ACS.

On the other hand, recent studies indicate that PLR can be a useful biomarker in the evaluation of patients with ACS, since a higher PLR ratio seems to be associated with worse cardiovascular outcomes and greater disease severity, as well as being associated with a greater extent of the coronary lesion in patients with ACS.^2^^,^^5^^,^^6^ Although PLR has lower sensitivity, specificity, and predictive value for the prognosis of MACE after PCI than NLR, both markers increased in patients with ACS 24 h after PCI and then decreased in the 30 days after the operation, which reinforces the importance of the concomitant association of the two ratios for stratifying cardiovascular risk and clinical decision-making.

## Conflicts of interest

The authors declare no conflicts of interest.
